# Supplementary material for: Outcome measures for young people with adolescent idiopathic scoliosis: A qualitative exploration of healthcare professionals’ perceptions and practices
Source: PLoS One. 2024 Jan 26;19(1):e0297339. doi: 10.1371/journal.pone.0297339 (PMC10817127; doi:10.1371/journal.pone.0297339)
Supplement: S1 Appendix — (PDF) [file pone.0297339.s001.pdf]

Topic guide of using Outcome measure for individuals with AIS

Ethics Statement/ instructions

Firstly, I would like to thank you for participating in this interview. My name is {Samia}, a PhD student from University of Birmingham. I realize that you are busy, and I appreciate your time to participate in this study. This interview aims to explore clinical use of outcome measure (OM) for Adolescent with Idiopathic Scoliosis, including any thoughts on barriers and enablers.

You were selected because you have a clinical or research experience in managing individuals with AIS. Please feel free to express your opinion and views clearly and in detail. There are no right or wrong answers; I am interested in your thoughts and experience. Just a reminder that this interview is being audio/video recorded. All information shared will be kept confidential and anonymised. Your name will not be used for the analysis and will be replaced by a code number. Before we start do you have any questions?

Introductory Questions

Would you please share with me your clinical/research experiences. (Years of experiences with AIS, clinical posts, setting, frequency of dealing/treating individuals with AIS).

Talking about outcome measures for individuals with AIS, could you please tell which questionnaire or OM you use in practice for AIS? (What type of OM you are using? Why? How often you use it? When do you use OM?)

Questions

Could you tell me what influences your choice of an OM when managing individuals with AIS? When you decide to choose OM/questionnaire what do you look for ?

- What about the characteristics of OM?
- How the characteristics of individuals with AIS influences your choice of OM?
- Could you perhaps tell me what you think is the most important factor that influence you to select OM for individuals with AIS?
- Thinking about the time constrains? How it affects your choice of OM?

From your point of view, how do different types of OMs other than PROMs be developed to enable its use in OM?

- Could you think of any example of OM? Consider performance and patient reported measures separately
- Have you use any experience to use performance-based OM?
- How do you think performance-based measures are different from other OMs such as PROMs?
- What could be done to enhance the use of performance-based measures in AIS?

What do you think are the limitations/barriers to using different types of OM in individuals with AIS?

- How the time constrain has an effect on your choice of OM?
- Could you think of other barriers? Recourses available?

Finally, is there anything connected to OMs used in AIS we haven't talked about and you think it's useful to bring it up now.
