# Supplementary material for: Outcome measures for young people with adolescent idiopathic scoliosis: A qualitative exploration of healthcare professionals’ perceptions and practices
Source: PLoS One. 2024 Jan 26;19(1):e0297339. doi: 10.1371/journal.pone.0297339 (PMC10817127; doi:10.1371/journal.pone.0297339)
Supplement: S2 Appendix — (PDF) [file pone.0297339.s002.pdf]

Table 2: Theme, subthemes, codes		
**Theme**	**Subthemes**	**Codes "Participants quotes"**
**Current practice**		
Routine practice	British spine registry system (BSR)	"Both trusts also expect our patients to participate in the British Spinal Registry, BSR. But I have to be honest, I've never seen what outcome measures or questions they're given in regard to the BSR, so that's probably something I do need to look into" P3
Personal evaluation	Subjective assessment	"This is what I was nervous of, I don't think I'm going to be very helpful. It would be subjective markers really. So if a patient's coming to me… so really there is, I'm just thinking like the last child who came and saw me. He was about 16. His goal was to… he was very conscious of his scoliosis. His goal was just to have better posture. So each time I'd ask him how's he feeling and the last time he saw me, he was like yeah I feel like I've got good postural awareness. I feel like I know how to get myself back into midline. I'm not getting any back pain and I'm doing everything I want to do. So he was like I don't think I need to do anymore. So very subjective." P2
	Use patients goals and needs	" Just with specific goals, really. So, we'd use patient-specific goals to identify what they want to achieve, what they're not able to do, and then use that just as a specific measure: measure as to how much they're improving"
	Percentage of improvement	" Sometimes we will use, like, a percentage of patient-reported… patient percentage improvement of how they feel they're doing with regards to returning to their normal functions or, yeah, how they're managing different episodes of flare-ups and things like that " P5
	Patient rate pain and function level	But really, I'm probably getting them to rate their pain scale on like a one to 10 maybe. I am also getting them, after a bit of physio, getting them to rate how much better they're feeling. So out of 100% but no formal outcome measures are being used in our department at the moment." P2
Monitoring outcomes	See patterns at long terms	"If a patient, for example, is being monitored for a few years, then prior to proceeding to need surgery, we could have maybe three years of data and seeing how the results have deteriorated or maybe not deteriorated, but we would see more of a… be able to see a larger pattern"P1
	Good scope of patient's status	"Whereas here we don't do that currently, we just do one pre-operatively. Obviously that wouldn't give us such a good scope and understanding of how they may feel pre-operatively or whether there has been a decline in how they feel." P1
	Quality of life measures important to children	"There are two or three aspects of the patient care. One of them is the clinical. The second is radiologic and the third is health related quality of life. I feel that the radiologic measures we just measure, so we don't need… it should ideally be involved in the outcome measure, but it doesn't have to be. Clinical assessment is clinical assessment. I think the outcome measure is most important for capturing the health-related quality of life." P6
**Barriers to use outcome measures**		
Priority and support	Difficult to implement	"Outcome measures have always been something that we want to get better at. But it's very difficult to implement that, people's compliance with it, get them trained up on it. Remember to do it when you're

		discharging a patient. Outcome measures are just notoriously difficult to put into place to improve compliance”. P2
	Focus on providing care	“Your clinic slot is only 4 hours, so that's half the time filling out questionnaires. That means that's half the time that you're not seeing patients. Of course, as a clinician, although it's nice to be involved in research, our primary focus is patient care, so it's a resource issue.” P4
Practical challenges	Did not inform practice	“Whereas with physio a lot of kids will have scoliosis and not need physio at all and they just crack on. So it doesn’t mean to be as formal because it’s not kind of make or break.” P2
	PROMs are inappropriate	“I mean it is quite a lengthy questionnaire. It is a number of pages and they, especially our young adults, the AIS patients, when they’re coming through, they have a number of different forms that they have to fill out for the trust. They are getting given four or five different pieces of paper to fill out and that all needs to be done before they come in for a clinic appointment”. “I think some of the language in it is quite outdated in comparison to language that we may use now” P1
	No time	“so, filling out these forms takes a long… I mean takes a reasonable amount of time. Then you'd have to add up the scores and then put it on the database, so that whole process could take, I don't know, 10, 15 minutes. So, it's just not practical to do it for a clinic of, I don't know, 10 patients, because suddenly you've got 110 minutes there. That's nearly 2 hours. Your clinic slot is only 4 hours, so that's half the time filling out questionnaires. That means that's half the time that you're not seeing patients” P4
	BSR is not user friendly	“The Amplitude system is not the most user friendly to get at and I don’t bother to look at it because I’m too busy. And I generally don’t know what the outcome measure tells me.” P3
Patient challenge’s	Potential invalidity	“Or you’re asking them questions and they don’t understand the terminology of the question, particularly with the younger teens. I think some of those things are true. So either you get what the parent thinks, which is not actually valid data or they kind of guess because they’re not quite sure. ..I think as a younger adolescent, you’re having a parent talk it through and then also if you’re talking it through, then you naturally may bring in your own judgement or suggestion of what something could be, which again, takes away the validity of it because one understanding, how they interpret it”P1
	Patient unwilling to participate	“Yeah, I think some patients don’t want to participate, so that’s automatically like a barrier.. again, we’re looking at a cohort of patients that don’t always want to engage. So that automatically you’ve got a barrier because they don’t want to engage with the research” P1
	No discharge assessment	“So sometimes you don’t ever get that formal discharge session anyway. Because you might get the mum just saying, oh we don’t need physio anymore, they’re better, so we’re not going to take them. So then you never get that formal goodbye and you don’t get that final outcome measure.” P2
Knowledge, education and perceived value	Lack of knowledge	“That is very nice that that percentage is 32%, but what does that mean? Is that a good thing or a bad thing? I don’t know. So definitely an area where I need to increase my knowledge if we are going to be utilising it more. But as I don’t see it in practice very often, I haven’t felt that it was something that was necessary for me to put the time in to learn what it was.”P7
	Questioning importance	“I think if we were to implement some sort of outcome measure, generally, there’s quite a lot of effort behind why it’s important and how it will improve my life, how it will improve my patient’s lives, you know. It’s no good doing it just because it makes a paper.” P3

	Never reviewed practice	"When I was an outpatient sister I used to hand them out and make sure that they had them with the research nurses. And then when I became the spinal clinical nurse specialist again, it was another one that, I guess, yeah, I probably never reviewed the practice." P1
	Professionals do not want to change	"Someone, like me to change what they do to a different thing, can take quite a lot of education. Just because everybody does what they do, they don't want to change." P3
**Facilitators to use outcome measures**		
Good exiting measure	Sufficient psychometric properties	"So, typically, ideally, you'd want the perfect one to have… it would be valid, it would be reproducible, it would be reliable, but you also want one that can be compared across different… the SRS-22 is used frequently in research studies, and it's published in research papers. Therefore, it makes sense to use that one because it's, in the body of literature, recognised as an acceptable form of scoring that you can then use to compare with other papers. " P4
	Updated and modernised	" I think if we had a different type of questionnaire that was more able to engage the appropriate population and updated, easy to understand terminology, I think we would get more accurate data and also better buy-in as well from the patient cohort that we actually want to treat. We don't want to treat the parent; we want to treat the young person" P1
	Relevant	"I said to you, I would group the patient. If you're looking at scoliosis, I would look at adolescent idiopathic, versus neuromuscular, versus syndromic. Then I might choose an outcome measure that would be more relevant to that particular group of patients". P4
	Personalised	"I think if we're going to measure outcome, that has to be front and centre. That needs to be one of the most important things. But it also then has to be personalised to that individual." P3
	Comparable and internationally acceptable	"The SRS-22 is used frequently in research studies, and it's published in research papers. Therefore, it makes sense to use that one because it's, in the body of literature, recognised as an acceptable form of scoring that you can then use to compare with other papers." P4
	Simple and easy	" They want colour, they want pictures maybe to understand as well. So say something simple as pain, you know, you absolutely can do… we will use smiley faces in clinical practice for patients to be able to understand." P1
Priority and support	Research department	I think that's why it's really important to have a vast research department that can support with that and I think even more so… obviously we all want to give evidence based best practice, don't we? And we all want to know what we are doing is clinically the best treatment for our patients. We want to also know our long-term outcomes because that's another thing, we discharge this patient cohort after two years and just think they're going to be fine."P1
	Scoring system	"But it's good if we have a scoring system, if you were to compare either two treatment modalities or we want to look at the outcome of a particular operation or whether that particular operation works. That's important. So scoring system is good from the research point of view. From the clinical point of view, the score domains are useful to identify any particular problem with the patient, so mental health, cosmetic issues, pain, things like that" P6

Fit into practice	Show difference in practice	“ The other side of it is it has to be easy to use, understandable, fit into clinical practice, not hold up my clinic and be something that means something to me, and means something to the individual. And it’s finding the balance between the two. I think the ideal thing” P3
**Barriers to use performance measures**		
Perceived value	Patient reported measure more important	“Patient reported… patient reported would definitely be more important. I think that my different patient with scoliosis, some would want to be able to do 100 push ups, and some just want to be able to take the dog for a walk and walk over a stile.” P2
	Performance differs across individuals	“Yeah absolutely. It would have to be quite… it would be really difficult to know how to structure that because like I say, some people’s performance is significantly different to others. So if we were just talking about can they, I don’t know, put their own shoes and socks and lean over? Could they sit to stand, could they get in and out of a bath? That would be helpful but very much an acute post-op situation.” P2
Practicality	Need physical space	“I think if we’re thinking about some sort of physical activity, then the problem we’d have with AIS, is physical space. I can do a paper form in clinic because I give it to the kid who is sitting on the chair. But if I want someone to, I don’t know, walk them down the corridor 20 times, or whatever it may be, it’s the practicalities of how you do that and how I do it with 30 children at the same time essentially.”P3
	Time	“That would be the thing for me, is if I was going to have something that wasn’t a PROM, how quickly could it be done, who would do it and where would they do it?” P3
Knowledge and experience	Don’t know about it	“So physically, yeah, objectively, rather than the patient. I don't know, probably not so useful. I wouldn't have thought so, in this kind of population. Obviously, we look at range of movement in all of these patients, so we would use range of movement as a guide, but probably not so useful. I would say with these ones, from my experience, it's much more about the patient-reported ones, and how they're feeling, and how they can manage, how they can cope.” P5
	Not used to use	“ Maybe it's just I'm not familiar with it, and it's not regularly used.” P4 “They do this quite a lot don’t they, with shoulders, hips and knees and other elements, don’t they? But I have to be honest, I haven’t seen it routinely used within adolescent spinal deformity at all. So yeah, I know obviously within other fields of orthopaedics, it is something that’s commonly quite used.” P1
	Performance indicators are unknown	“The point I'm trying to make is which one do you place the most emphasis on? There are so many different performance indicators that you could use, that one could potentially use.

		Which one would you place emphasis on? We just tend to ask the patients whether they can do the things that they enjoy doing, which is that… I guess they then place emphasis on what they think is most important to them." P4
**Facilitators to use performance measures**		
Practical considerations	Time	"I guess it's just then putting it into practice isn't it? Formulating it as part of your… or when you do a clinical assessment, you're always going to achieve these markers. But I think you do that naturally within consultation, so maybe it wouldn't add that much more to your time if done in an appropriate manner and if it was very simplistic as well. If we were just looking at" P1
	Relevant	" You have to decide what is… if you're going to use it across multiple sites, you need to pick a universal measure that's relevant to everyone. I'm not familiar with one yet that would have the same applicability." P4
	Cost	"I think if there was any sort of outcome measure that made a difference… so, there's this question about whether AIS surgery actually makes a different. Is it worth doing it? Is it cost-effective, etc.?" P3
	Acceptability	" I think the other thing is… going back to the acceptability. My research is in surface shape, surface shape means that teenagers have to expose themselves, to a degree. If you're going to have an outcome measure related to something like that, how do you do that and maintain dignity? Maintain self-protection, child protection, all of those issues, all have to be figured in as well." P3
